# A Novel Fluorescence Sensor for Iodide Detection Based on the 1,3-Diaryl Pyrazole Unit with AIE and Mechanochromic Fluorescence Behavior

**DOI:** 10.3390/molecules28207111

**Published:** 2023-10-16

**Authors:** Lili Deng, Jian Xiong, Wenqin Liu, Lixue Wu, Huiyi Hu, Jiaqing Wu, Yue Liu, Lide Yu, Yuling Zhou, Wenjun Gao, Haifeng He, Weiyan Yin

**Affiliations:** 1 School of Pharmacy, Jiangxi University of Chinese Medicine, Nanchang 330013, China; m15177905887@163.com (L.D.); 20040798@jxutcm.edu.cn (J.X.); 19970251@jxutcm.edu.cn (W.L.); wulixue@jxutcm.edu.cn (L.W.); huhuiyi@jxutcm.edu.cn (H.H.); 201901006097@jxutcm.edu.cn (J.W.); 201901012012@jxutcm.edu.cn (Y.L.); 19910244@jxutcm.edu.cn (W.G.); 2 Jiangxi Provincial Engineering Research Center for Waterborne Coatings, School of Chemistry and Chemical Engineering, Jiangxi Science and Technology Normal University, Nanchang 330013, China; hehf0427@jxstnu.com.cn; 3 Key Laboratory of Tropical Fruits and Vegetables Quality and Safety for State Market Regulation, Hainan Institute for Food Control, Haikou 570314, China; 4 Hubei Key Laboratory of Biomass Fibers and Eco-Dyeing & Finishing, School of Chemistry and Chemical Engineering, Wuhan Textile University, Wuhan 430073, China; hpywy2006@163.com

**Keywords:** TPA-CDP, triphenylamine, 1,3-diaryl pyrazole, sensor, mechanochromic fluorescence, AIE, XRD

## Abstract

A D−A type of luminophore, TPA-CDP, was designed and synthesized by using triphenylamine (TPA) as D (electron donor), 1,3-diaryl pyrazole with cyano groups (CDP) as A (electron acceptor) and employing a cyanovinyl segment as a recognition group. Firstly, TPA-CDP demonstrates effective fluorescence quenching as a sensor for I^−^ by the nucleophilic addition reaction of the cyanovinyl segment with a high level of sensitivity, selectivity and a low determination limit of 4.43 μM. Interestingly, TPA-CDP exhibited an AIE phenomenon with the *f_w_* value reaching 50%. In addition, TPA-CDP displayed distinct mechanochromic fluorescence behavior with 70 nm red shift, which was observed over four repeated cycles. Furthermore, the mechanochromic fluorescence behavior of TPA-CDP, as observed in powder XRD experiments, was found to be associated with the morphological transition from a crystalline state to an amorphous state. These results confirm the significant potential of CDP as a powerful electron-deficient component in the creation of D−A-type mechanochromic fluorescence materials and biosensors for detecting I^−^.

## 1. Introduction

Iodide has garnered significant attention in the academic community due to its pivotal role in various biological activities associated with thyroid and neurological functions [1,2,3]. Both an excess and deficiency of iodine can lead to the development of hypothyroidism [4,5], neurological disorders [6], endemic goiter [7], cretinism, and thyroid lesions [8]. The implementation of real-time monitoring of iodide could potentially facilitate the identification and tracking of these disorders in a robust manner. Furthermore, the synthesis of chemicals, such as drugs and dyes, utilizing elemental iodine [9] has been explored. In addition, since ^129^I is a long-lived radioisotope that is necessary for nuclear weapon testing, it is a major contributor to environmental pollution through various sources [10,11]. Considering the above findings, it is imperative that new probes are developed that selectively recognize iodide over other anions. In contrast to other halides, iodide with its large size, weaker basicity and low charge density, shows weak binding ability, which makes it a challenging task to design iodide-selective probes. For the determination of iodide, a variety of methods have been used, such as neutron activation analysis, capillary electrophoresis, iodide-selective electrodes, atomic absorption spectroscopy (AAS) [12], inductively coupled plasma mass spectrometry (ICP-MS), gas chromatography (GC) and electrostatic ion chromatography (EIC) [13,14,15,16]. Among all, ICP-MS is the most commonly used mass spectrometer. However, it requires sophisticated instrumentation as well as tedious determination procedures.

Recently, fluorescence analysis has become increasingly popular as a powerful monitoring tool for ion determination. A typical sensor generally consists of the following three components: the recognition group, the linker, and the fluorophore. Sensors assembled from these components usually exhibit no fluorescence or a changed fluorescence compared with the original fluorophore. Sensors with advantages in terms of sensitivity, interference-free ability, and low LOD due to the recognition group can react or coordinate according to the molecular molar ratio [17,18,19].

To date, a variety of materials have been reported for detecting and tracking I^−^ [20,21,22,23,24]. It is still necessary for synthetic chemists to develop probes with novel modes of action despite impressive advances in these probes due to limitations such as the high determination limit and low sensitivity of these probes. However, a new category of fluorescent materials known as probes with aggregation-induced emission (AIE) has recently emerged. These materials possess emission properties that differ from conventional fluorophores, as they are non-emissive in solutions due to aggregation-caused quenching (ACQ) but become emissive upon aggregation due to aggregation-induced emission (AIE) [25,26,27].

Over the past decade, there has been extensive utilization of a diverse range of AIE fluorogens as chemo/biosensors for the detection of various analytes [28,29]. These AIE-active fluorescent assays, which rely on the stimuli-responsive aggregation and emission enhancement behavior of the fluorogens, have demonstrated high sensitivity and selectivity [30]. Notably, AIE-active probes exhibit remarkable adaptability to different working environments, including solutions, aggregates, and solid states, enabling the identification of analytes even under challenging conditions [31]. Additionally, probes with AIE properties can be used for bioimaging and biosensing in vivo due to their bright fluorescence in aggregation state [32]. Furthermore, it goes without saying that molecules exhibiting an AIE effect make excellent candidates for high-performance mechanical force-responsive luminous materials [33]. Noticeably, as smart functionalized materials, mechanochromic materials with luminescent colors that change under mechanical stimuli have attracted a lot of attention [34,35,36]. In spite of this, there is still a lack of mechanochromic fluorescent compounds that are AIE-active [37].

**Scheme 1:**
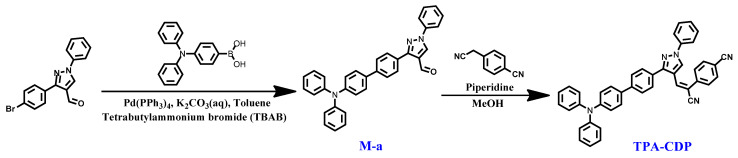
Synthesis of probe TPA-CDP.

Conjugated donor-acceptor (D-A) structures exhibiting push-pull effects are commonly found in fluorescent molecules, often adopting twisted molecular conformations. These conformations play a crucial role in modulating the fluorescence sensitivity to aggregation and mechanical forces [38,39], thereby facilitating the emergence of aggregation-induced emission (AIE) effects. Additionally, the cyano group (CN) is frequently employed in biological systems as a means to manipulate molecular geometry and electron distribution due to its electron-withdrawing capabilities [40,41,42]. In contrast, the utilization of triphenylamine (TPA) fluorogens with twisted molecular conformations is sought after for the construction of emission materials with high efficiency [43,44,45]. Therefore, the present study focuses on the development of a D−A-type probe, namely TPA-CDP, which incorporates 1,3-diaryl pyrazole with cyano groups (CDP) as the electron acceptor (A), triphenylamine (TPA) as the electron donor (D) and employs a cyanovinyl segment as the recognition group (Scheme 1). The TPA-CDP probe demonstrated notable selectivity and sensitivity towards I^−^, as demonstrated by the reaction between the cyanovinyl segment and I^−^, resulting in a remarkably low limit of determination of 4.43 μM. The observable change in color from colorless to yellow enhances its ease of detection by visual observation. Consequently, it holds great promise as a potential biosensor for I^−^ in practical applications. Additionally, the TPA-CDP probe, with its AIE-active properties, exhibited a vibrant yellow fluorescence. Notably, it displayed reversible and bathochromic mechanochromic fluorescence characteristics.

## 2. Results and Discussion

### 2.1. Optical Response of the TPA-CDP Probe

**Figure 1:**
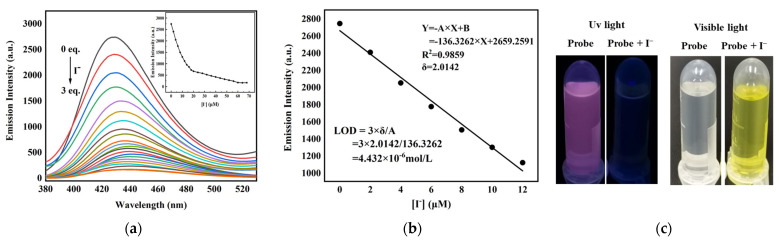
(**a**) Fluorescence titration spectra of TPA-CDP (20 μM, λ_ex_ = 345 nm, λ_em_ = 431 nm) in THF; (**b**) the LOD of TPA-CDP against I^−^; (**c**) pictures of the solution with I^−^ (3 eq) addition before and after.

With regard to TPA-CDP, based on cyanovinyl structural variation triggered by I^−^, the performance of TPA-CDP using a 4-cyano-4′-cyanophenylmethylene-1,3-diarylpyrazole acceptor unit a sensor to detect I^−^ was investigated. Fluorescence titrations were conducted using 0 to 70 μM of I^−^. As depicted in Figure 1a, the fluorescence intensity gradually decreased upon the addition of I^−^. Then, a plateau was observed upon the addition of I^−^ at 70 μM. As indicated by the discernable variations in fluorescence intensity, TPA-CDP was able to efficiently detect I^−^. In response to the good linear relationship between fluorescence intensity versus [I^−^] (R = 0.9859) (Figure 1b) between 0 and 12 μM, the determination limit (LOD) for TPA-CDP was calculated to be 4.43 μM using Equation (1.2) (Supplementary Materials) [46,47]. Additionally, the combination of TPA-CDP with I^−^ resulted in noticeable changes in color by the naked eye, from colorless to yellow (Figure 1c). Moreover, TPA-CDP could quickly detect I^−^ (only 0.5 min, Figure S1). Its rapid and macroscopic detection properties are suitable for recognizing real samples with portable devices.

**Figure 2:**
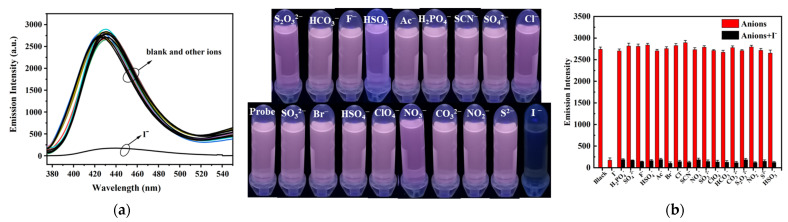
(**a**) Fluorescence spectra of TPA-CDP in THF (20 μM) upon addition of 3 equiv. of each anion; (**b**) histogram of the emission intensity of TPA-CDP in THF solution (20 μM) with various competitive anions (3 eq) and I^−^ (3 eq).

Selectivity is considered a significant symbol for practical applications when the sensor is capable of detecting multiple anions, including H_2_PO_4_^−^, SO_4_^2−^, F^−^, HSO_4_^−^, Ac^−^, Br^−^, Cl^−^, SCN^−^, NO_2_^−^, SO_3_^2−^, ClO_4_^−^, HCO_3_^−^, CO_3_^2−^, S_2_O_3_^2−^, NO_3_^−^, S^2−^, and HSO_3_^−^. Therefore, the binding ability of TPA-CDP with I^−^ was further validated by recording fluorescence spectra in presence and absence of I^−^ in THF. A fluorescent emission of the free sensor TPA-CDP was observed at 431 nm with excitation 345 nm in THF. Figure 2a shows that other competitive anions have an insignificant effect on the fluorescence intensity of TPA-CDP. Additionally, in presence of I^−^, the pink solution of TPA-CDP changes to colorless under UV light.

An effective fluorescent probe must select for a particular analyte in the presence of multiple competing ions. Thus, to investigate the TPA-CDP’s selectivity, competitive experiments were conducted in the presence of 3 equiv. of I^−^ and 3 equiv. of other tested anions. Figure 2b illustrates that the emission intensity of TPA-CDP in the presence of I^−^ was not significantly affected by other competitive anions, revealing TPA-CDP’s excellent selectivity towards I^−^. As demonstrated by these findings, TPA-CDP responded specifically to I^−^. With its low LOD, remarkable sensitivity, and selectivity, the TPA-CDP probe exhibits promising potential for practical utilization in the detection of I^−^.

### 2.2. Aggregation−Induced Emission (AIE) Characteristics of TPA-CDP

**Figure 3:**
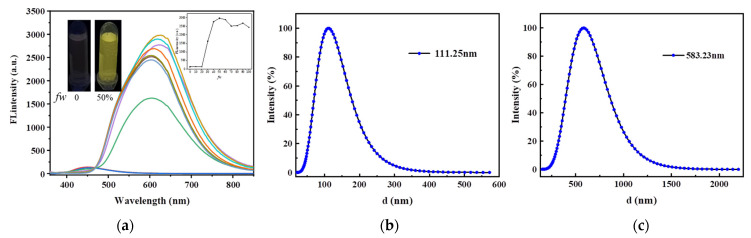
(**a**) Emission spectra (λ_ex_ = 345 nm) of the dilute solutions (20 μM) of TPA-CDP in DMF–H_2_O mixtures with various *f_w_* values (0–100%); size distribution measured by DLS of the TPA-CDP in DMF–H_2_O mixtures with 50% (**b**) and 100% (**c**) water content. Concentration: 20 μM.

**Figure 4:**
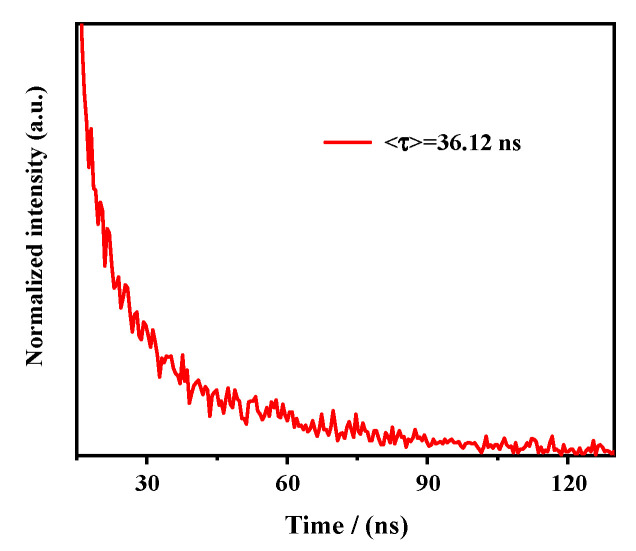
Decay curves of TPA-CDP (λ_em_ 345 nm, 20 μM) with *f_w_* values of 50%.

AIE properties of TPA-CDP were initially investigated by UV–visible absorption and photoluminescence spectroscopy. TPA-CDP showed little variation in absorbance spectra when diluted with different volumes of water (*f_w_*) DMF/H_2_O mixtures, and an electronic transition could be responsible for the absorption peaks below 450 nm. Furthermore, level-off tails, which are linked to the light-scattering phenomenon caused by nano-aggregates, were detected in the longer wavelength range as the *f_w_* value increased (Figure S2), thereby suggesting the presence of nano-aggregates [48,49]. Figure 3a illustrates that TPA-CDP, dissolved in a DMF solution (20 μM), showed minimal luminescence when exposed to UV light at 365 nm, with a very weak emission band (λ_max_) observed at 451 nm. As *f_w_* increased to 30%, a new emission band was observed at 605 nm, and the yellow luminescence was observed under UV (365 nm) irradiation. Further enhancement of the luminescence occurred with *f_w_* at 50% with a corresponding Φ_F_ of 20.54%, and then the fluorescence intensity weaken when *f_w_* increased due to the large diameter of the aggregates. Furthermore, the nano-aggregates of TPA-CDP were confirmed by dynamic light-scattering (DLS) measurements with *f_w_* values of 50% and 100% (Figure 3b,c). The diameter of the aggregates were 111.25 nm and 583.23 nm, respectively. Interestingly, a DMF solution (20 μM) of the previously reported probe [32] showed a strong emission band (λ_max_) of 447 nm with the solution emitting a blue luminescence under UV light at 365 nm, while TPA-CDP (this work) showed negligible emission. Then, the previously reported probe showed a new emission band (λ_max_) at 626 nm as the *f_w_* increased further to 90%, and the blue luminescence changed to orange-yellow luminescence under UV irradiation (365 nm). However, in this work, TPA-CDP showed negligible emission with *f_w_*=0; as *f_w_* increased to 30%, a new emission band was observed at 605 nm, and the yellow luminescence was observed under UV (365 nm) irradiation. According to these results, luminophore TPA-CDP exhibits an AIE behavior due to the restriction of intramolecular rotation in the compound, while the previously reported probe exhibits an aggregation-induced emission enhancement (AIEE) behavior. In Figure 4, probe TPA-CDP, with an *f_w_* of 50%, showed a 36.12 ns average luminescence lifetime, indicating that the TPA-CDP luminophores with a *f_w_* of 50% exhibited fluorescence behavior [50,51]. Interestingly, TPA-CDP is derived from the work in [32] by changing the electron-withdrawing ability of the cyanovinyl group to regulate the electron cloud distribution of the structure. The structures in the literature exhibit AIE enhancement, while the typical AIE features are shown in probe TPA-CDP. The findings indicate that modulation of the electron cloud distribution governs the luminescent properties.

### 2.3. Crystal Solid−State Emission of TPA-CDP Compounds

**Figure 5:**
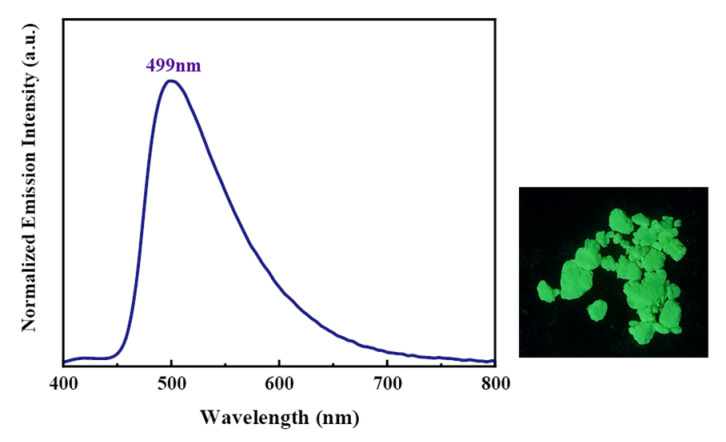
Crystal solid-state emissive spectra of TPA-CDP; the corresponding crystal solid-state fluorescence image under 365 nm UV light.

A room-temperature study of the fluorescence properties of the D−A-type luminophore TPA-CDP was performed. Figure 5 shows that the D−A-type luminophore, TPA-CDP, emits at 499 nm in the solid state, with an average emission lifetime, ˂τ˃, of 10.38 ns (Figure S3), corresponding to green fluorescence (Φ = 28.43%) under UV irradiation at 365 nm. Moreover, thermogravimetric analysis (TGA) of TPA-CDP revealed its thermal stability. Figure S4 illustrates that TPA-CDP exhibited good thermal stability, with 4% weight loss between 100 °C and 400 °C.

**Figure 6:**
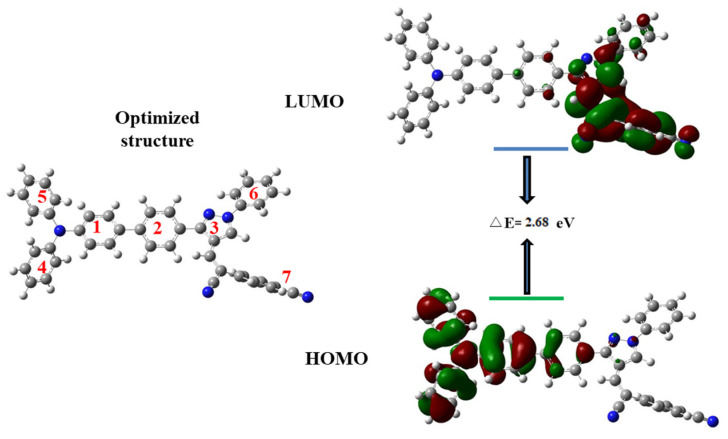
Optimized geometry and contour plots of the electron density distribution of the HOMO and LUMO for TPA-CDP.

In order to comprehend the characteristics of the ground and excited electronic states, density functional theory (DFT) calculations were performed on TPA-CDP at the B3LYP/6-31G* level using Gaussian 09 [52]. As depicted in Figure 6, in the optimized structures of TPA-CDP, the aryl rings 1, 2, and 3 are almost coplanar, and the aryl rings 4, 5, 6, and 7 are twisted away from the backbone, especially aryl rings 4, 5, and 7. In addition, π-electrons in the (highest occupied molecular orbital) HOMO of TPA-CDP are scattered on TPA, whereas in the (lowest unoccupied molecular orbital) LUMO, π−electrons are localized on CDP. In this case, the calculation results clearly showed the ICT situation. The relative energy bandgaps between the HOMO and LUMO are also presented in Figure 6.

### 2.4. Contrasting Mechanochromic Fluorescence Behavior of TPA-CDP Compounds

**Figure 7:**
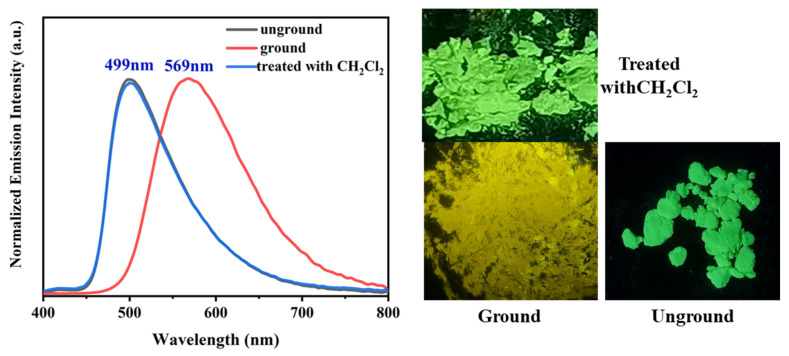
Solid-state emissive spectra of TPA-CDP under different conditions with λ_ex_ 345 nm; fluorescence image of the unground sample of TPA-CDP, ground sample of TPA-CDP, and the ground sample of TPA-CDP after treatment with CH_2_Cl_2_ under 365 nm UV light.

The high−contrast mechanochromic fluorescence displayed by luminophores has garnered significant interest in the fields of sensing and security encryption applications. Given that AIE-active molecules exhibit a heightened response to external stimuli due to the chromic effect, an experiment was conducted to investigate the mechanochromic fluorescence of the luminophore TPA-CDP. As previously mentioned, the green fluorescence of crystals solid TPA-CDP was observed at 499 nm (Figure 5) with an average lifetime of 10.38 ns (Figure S3). In an interesting observation, after grinding the crystal solid sample of TPA-CDP, the maximum emissions were observed at 569 nm with a red shift of 70 nm, and the ground powder emitted a yellow fluorescence (Figure 7) with an average lifetime of 59.45 ns (Figure S3).

Furthermore, after fumigating the ground powder with CH_2_Cl_2_ vapor for 1 min, a rapid return to the original color (green, Figure 7) of fluorescence occurred, and the average lifetime reduced to 12.31 ns (Figure S3). It follows that TPA-CDP has a reversible mechanochromic fluorescence behavior.

**Figure 8:**
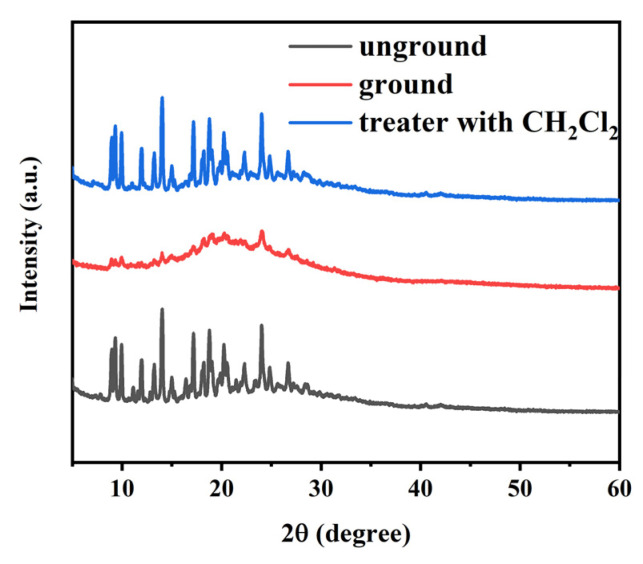
Powder XRD patterns of TPA-CDP in different solid states.

For a better understanding of the variation in the molecules responding to grinding, powder X-ray diffraction (PXRD) patterns were used to gain the mechanochromic fluorescence behavior (Figure 8). The pristine TPA-CDP exhibited multiple distinct diffraction peaks in its PXRD pattern, suggesting it is crystalline in nature without any grinding. Following the grinding process, the intensity of the diffraction peaks diminished or even vanished, implying that mechanical grinding resulted in an amorphous sample of TPA-CDP. Additionally, fumigating the ground sample with CH_2_Cl_2_ vapor for a duration of 1 min led to the restoration of the distinct diffraction peaks, closely resembling those of the unground sample, thereby implying the recovery of the crystal structure. Evidently, the reversible process of mechanochromic fluorescence matches the reversible phase transformation of the crystalline state to the amorphous state.

**Figure 9:**
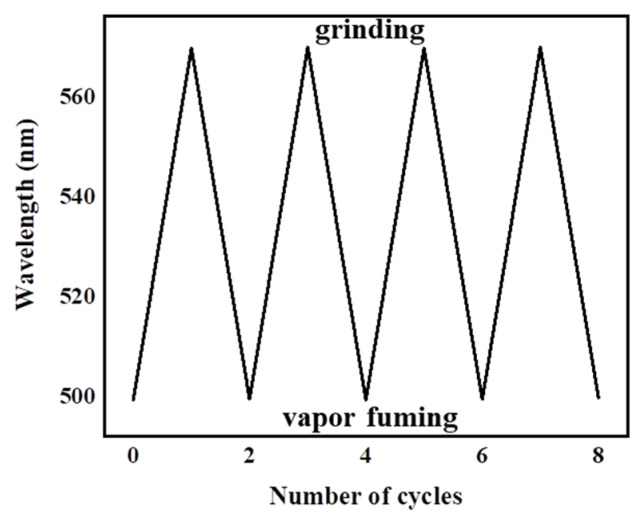
Repetitive experiment of the mechanochromic fluorescence effect for TPA-CDP.

Finally, the cycle of mechanochromic fluorescence behavior could be iterated four times, demonstrating the remarkable reversible mechanochromic fluorescence characteristics of TPA-CDP (Figure 9). Hence, TPA-CDP demonstrate a remarkable reversible mechanochromic fluorescence phenomenon.

## 3. Conclusions

Herein, we designed and synthesized a D−A-type solid crystal fluorophore based on 1,3-diaryl pyrazole containing TPA (D) and CDP (A and I^−^ receptor) units. AIE−active TPA-CDP demonstrates effective fluorescence quenching by means of its ability to sense I^−^ through the nucleophilic addition reaction of the cyanovinyl segment. Demonstrating through selectivity, competition, and titration experiments, TPA-CDP exhibits a high level of sensitivity and selectivity as a sensor for I^−^ with a low determination limit of 4.43 μM. Significantly, the color of TPA-CDP transitioned from colorless transparent to a vibrant yellow when I^−^ was introduced under visible light, indicating its potential as a naked-eye visual indicator for I^−^. Interestingly, when the *f_w_* value reached 50%, TPA-CDP exhibited an AIE phenomenon, which was attributed to the limitation of intramolecular rotation within the compound. Simultaneously, the TPA-CDP luminophore exhibited strong solid-state emissive properties in the green fluorescence range. TPA-CDP, with a 70 nm red shift, which has a planar structural organization, is sensitive to mechanical stimuli. The PXRD tests showed that the mechanochromic fluorescence of TPA-CDP was associated with a morphological transformation from a crystalline state to an amorphous state. Moreover, the TPA-CDP demonstrated reversible and bathochromic mechanochromic fluorescence phenomena, attributed to the transition from a crystalline to an amorphous state. Additionally, it exhibited a reversible mechanochromic fluorescence process resulting from the transformation from a crystalline to an amorphous state. The outcomes of this study provide a valuable reference for the development of exceptional mechanofluorochromic materials and the design of biosensors for the detection of I^−^.

## Data Availability

The data are available upon request to the authors.

## Supplementary Materials

The following supporting information can be downloaded at https://www.mdpi.com/article/10.3390/molecules28207111/s1, Figure S1: Effect of response time on the fluorescence intensity of TPA-CDP (20 μM) in the presence of I^−^; Figure S2: UV-–vVis spectra of TPA-CDP in DMF-–water mixtures with of varyingous water contents (0–100%). Concentration: 20 μM; Figure S3: Fluorescence decay curves of unground solid TPA-CDP (345 nm), ground solid TPA-CDP (345 nm), and ground solid TPA-CDP (345 nm) after treatment with dichloromethane; Figure S4: TGA thermograms of solid-state TPA-CDP; Table S1: The cartesian coordinates of the optimized geometry.

## Sample Availability

Samples of the compounds data are available from the authors.
