# Supplementary material for: A Novel Fluorescence Sensor for Iodide Detection Based on the 1,3-Diaryl Pyrazole Unit with AIE and Mechanochromic Fluorescence Behavior
Source: Molecules. 2023 Oct 16;28(20):7111. doi: 10.3390/molecules28207111 (PMC10609397; doi:10.3390/molecules28207111)
Supplement: Supplementary file 1 [file molecules-28-07111-s001.zip › molecules-2621453-supplementary.pdf]

Supplementary Information (SI)

A Novel Fluorescence Sensor for Iodide Detection Based on the 1,3-Diaryl Pyrazole Unit with AIE and Mechanochromic Fluorescence Behavior

Lili Deng [1,2,†], Jian Xiong [1,†], Wenqin Liu [1], Lixue Wu [1], Huiyi Hu [1], Jiaqing Wu [1], Yue Liu [1], Lide Yu [1,*], Yuling Zhou [3,*], Wenjun Gao [1], Haifeng He [2] and Weiyan Yin [4]

[1] School of Pharmacy, Jiangxi University of Chinese Medicine, Nanchang 330013, China; m15177905887@163.com (L.D.); 20040798@jxutcm.edu.cn (J.X.); 19970251@jxutcm.edu.cn (W.L.); wulixue@jxutcm.edu.cn (L.W.); huhuiyi@jxutcm.edu.cn (H.H.); 201901006097@jxutcm.edu.cn (J.W.); 201901012012@jxutcm.edu.cn (Y.L.); 19910244@jxutcm.edu.cn (W.G.)
[2] Jiangxi Provincial Engineering Research Center for Waterborne Coatings, School of Chemistry and Chemical Engineering, Jiangxi Science and Technology Normal University, Nanchang 330013, China; hehf0427@jxstnu.com.cn
[3] Key Laboratory of Tropical Fruits and Vegetables Quality and Safety for State Market Regulation, Hainan Institute for Food Control, Haikou 570314, China
[4] Hubei Key Laboratory of Biomass Fibers and Eco-dyeing & Finishing, School of Chemistry and Chemical Engineering, Wuhan Textile University, Wuhan 430073, China; hpywy2006@163.com
* Correspondence: 20020680@jxutcm.edu.cn (L.Y.); 18689983973@189.com (Y.Z.)
† These authors contributed equally to this work.

1. Experimental Methods

1.1. General Information

The starting materials and all reagents were obtained via commercial suppliers and then used without further purification. Various anions were prepared using NaH_2PO_4, Na_2SO_4, NaF, $NaHSO_4$, NaAc, NaBr, KI, NaCl, NaSCN, $NaNO_2$, Na_2SO_3, $NaClO_4$, $NaHCO_3$, Na_2CO_3, $Na_2S_2O_3$, $NaNO_3$, Na_2S, and $NaHSO_3$. TPA-CDP was synthesized according to the relevant literature [1]. All experiments were carried out under an argon atmosphere using standard Schlenk techniques. ^{1}H NMR (400 MHz) and ^{13}C NMR (100 MHz) spectra were recorded on an American Varian Mercury Plus 400 spectrometer (400 MHz). ^{1}H NMR spectra are reported as follows: chemical shift in ppm (δ) relative to the chemical shift of TMS at 0.00 ppm, integration, multiplicities (s = singlet, d = doublet, t = triplet and m = multiplet), and coupling constant (Hz). ^{13}C NMR chemical shifts were reported in ppm (δ) relative to the central line of the triplet for $CDCl_3$ at 77 ppm. Mass spectral data were recorded on a Bruker UltrafleXtreme MALDI-TOF-TOF mass spectrometer. Fluorescence spectra were recorded by a fluorescence spectrometer (FLS980, Edinburgh Instruments). The absolute fluorescence quantum yields were measured using a HAMAMATSU ABSOLUTE PL QUANTUM YIELD SPECTROMETER C11347. The fluorescence lifetimes were measured using an FLS980. XRD studies of TPA-CDP were recorded on a Shimadzu XRD-6000 diffractometer (Japan) (Cu Kα, 40 kV and 30 mA). UV–is absorption spectra of TPA-CDP were obtained on an Agilent 8453 UV/Vis spectrophotometer. Size distribution was measured using a BeNano 90.

1.2. Determination of the Detection Limit

The detection limit was calculated based on fluorescence titration as a function of the solubility of I^-. The fluorescence emission spectrum of the TPA-CDP free probe was measured 12 times to determine the standard deviation for a blank measurement. A linear plot was constructed with the average intensities plotted against the concentration of I^- ions to determine the slope. Using the slope, the detection limit was calculated from the following equation [2,3]:

$$\mathrm{LOD} = \frac{3\sigma}{\mathrm{K}}$$

where σ is the standard deviation of the blank solution and K is the slope between the intensity versus the sample concentration.

1.3. General Procedure for I^- Detection

Unless otherwise stated, the fluorescence of TPA-CDP (20 μM) reacting with iodine ions was determined in THF as follows. The stock solution (1 mM) of TPA-CDP was made by dissolving solid samples in DMF. In a test tube, 4 mL of THF and 100 μL of TPA-CDP stock solution were mixed; this was followed by the addition of an iodine ion sample solution. The final volume was adjusted to 5 mL with THF. After mixing, 3 mL of the reaction solution was transferred to a 1 cm quartz cell to measure the absorbance or fluorescence with $\lambda_{ex/em}$ = 345/431 nm and excitation and emission slit widths of 5 nm.

1.4. General Procedure for the Water/DMF Mixing Experiments

The procedure for preparing the water/DMF mixed solvent with varying volume ratios ranging from 0% to 100% is as follows. Eleven test tubes were labeled accordingly, representing the different ratios (0%, 10%, 20%, 30%, 40%, 50%, 60%, 70%, 80%, 90%, and 100%). In the first tube (0%), 10 mL of DMF was added. In the second tube (10%), 1 mL of water and 9 mL of DMF were added, followed by thorough stirring and allowing the mixture to stand. Similarly, in the third tube (20%), 2 mL of water and 8 mL of DMF were added, stirred well, and allowed to stand. This process was repeated for the subsequent tubes, with increasing amounts of water and decreasing amounts of DMF until the sixth tube (50%), where 5 mL of water and 5 mL of DMF were added and stirred well.

1.5. General Procedure for the Synthesis of TPA-CDP

The synthesis process of the intermediate M-a is as follows: Under an argon atmosphere, a mixture of 3-(4-bromophenyl)-1-phenyl-1H-pyrazole-4-carbaldehyde (981 mg, 3.00 mmoL), (4-(diphenylamino)phenyl)boronic acid (1.73 g, 6.00 mmoL), K_2CO_3 (1 mol/L, 5 ml, aqueous solution), tetrabutylammonium bromide (710 mg, 2.20 mmoL), and $Pd(PPh_3)_4$ (350 mg, 0.30 mmoL) were stirred in toluene (60 mL) at 80 °C. After the reaction was known to be complete by TLC detection, the mixture was extracted three times with 60 mL dichloromethane, and then the combined organic layers were washed with brine, dried (MgSO4), and subjected to reduced-pressure distillation. The residues were purified by column chromatography, affording a white solid product at a yield of 79.2%. ^{1}H NMR (400 MHz, $CDCl_3$): δ (ppm) = 10.20 (s, 1H), 8.66 (s, 1H), 8.00 (d, *J* = 8.2 Hz, 2H), 7.91 (d, *J* = 8.0 Hz, 2H), 7.81 (d, *J* = 8.3 Hz, 2H), 7.63 (t, *J* = 8.5 Hz, 4H), 7.50 (t, *J* = 7.4 Hz, 1H), 7.38 (dd, *J* = 12.8 5.3 Hz, 6H), 7.25 (d, *J* = 2.9 Hz, 3H), 7.15 (t, *J* = 7.3 Hz, 3H).

The synthesis process of TPA-CDP is as follows: Under an argon atmosphere, a mixture of intermediate M-a (491 mg, 1 mmoL), 4-(cyanomethyl)benzonitrile (142 mg, 1 mmoL,) and piperidine (0.1 mL) was stirred in dry methanol (15 mL) at room temperature for 2 h. After completion of the reaction, confirmed by TLC detection, the solid was filtered, washed with methanol, and dried. The solid was purified by column chromatography, affording a yellow solid product at a yield of 75.6%. ^{1}H NMR (400 MHz, DMSO-d_6): δ (ppm) = 9.21 (s, 1H), 7.96 (dd, *J* = 19.4 11.7 Hz, 7H), 7.80 (d, *J* = 7.8 Hz, 4H), 7.68 (d, *J* = 7.5 Hz, 2H), 7.61 (s, 2H), 7.45 (s, 1H), 7.34 (d, *J* = 6.8 Hz, 4H), 7.08 (d, *J* = 6.4 Hz, 8H); ^{13}C NMR (100 MHz, $CDCl_3$): δ (ppm) = 154.96, 147.82, 147.57, 141.46, 139.23, 138.39, 135.22, 133.76, 132.85, 129.88, 129.66, 129.49, 129.35, 127.71, 127.07, 125.93, 124.66, 123.65, 123.23, 119.78, 118.24, 118.03, 115.95, 112.16, 107.03. ESI-MS: m/z = 615.4 $[M]^+$.

1.6.1. H NMR and ^{13}C NMR spectra

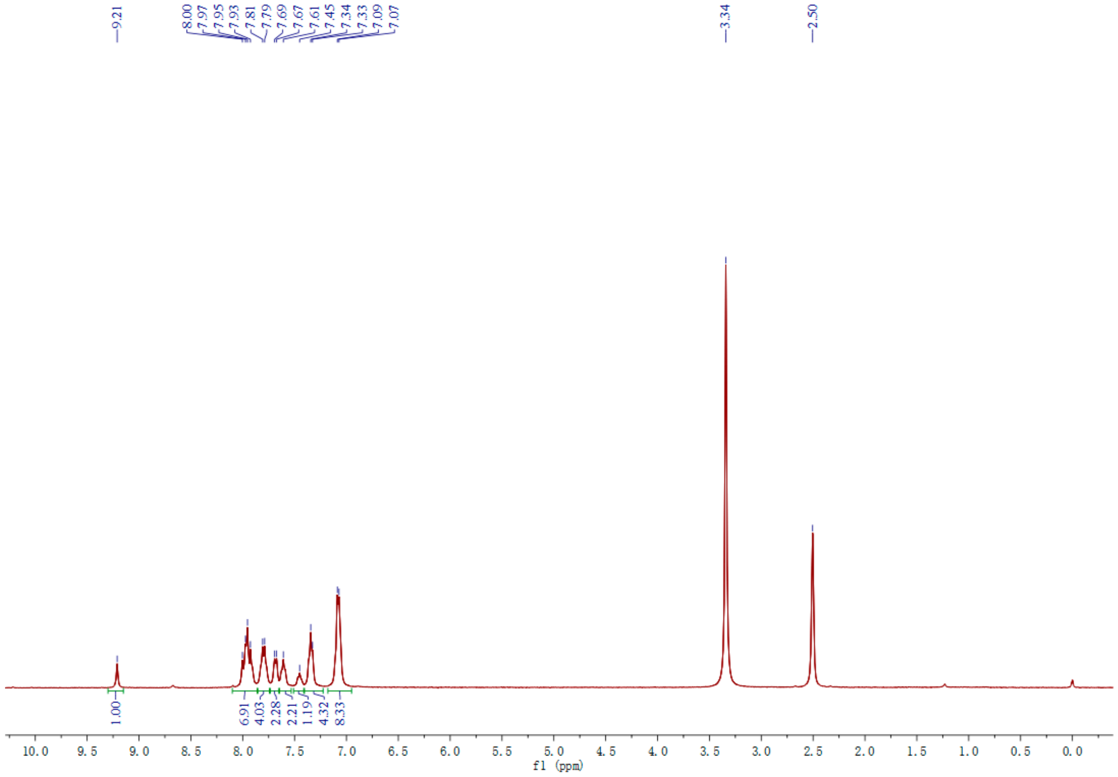

^{1}H NMR spectra of TPA-CDP.

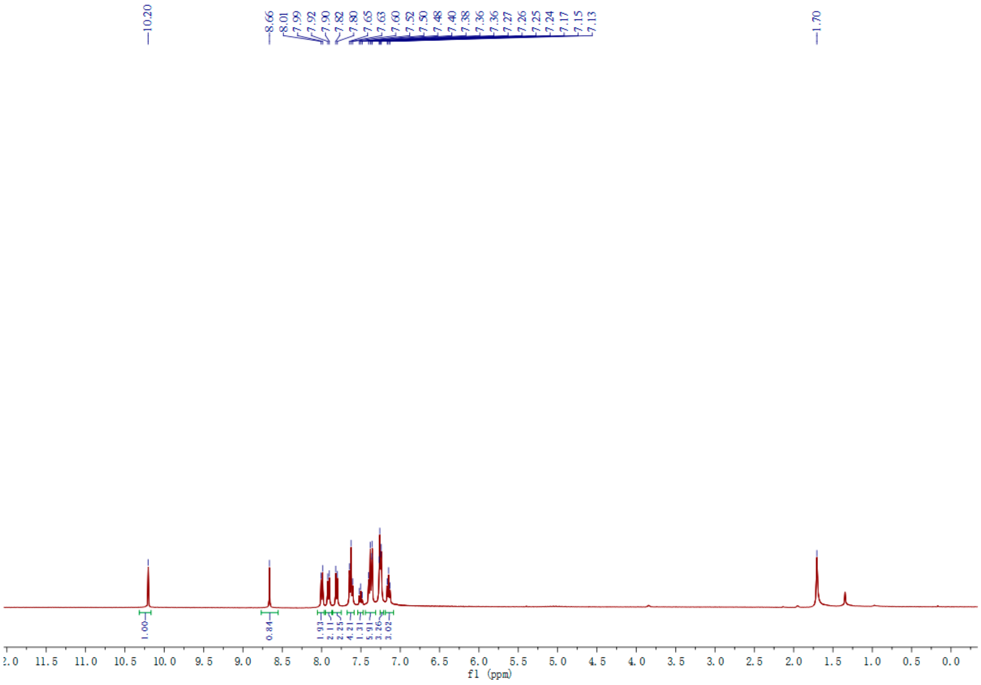

^{1}H NMR spectra of M-a.

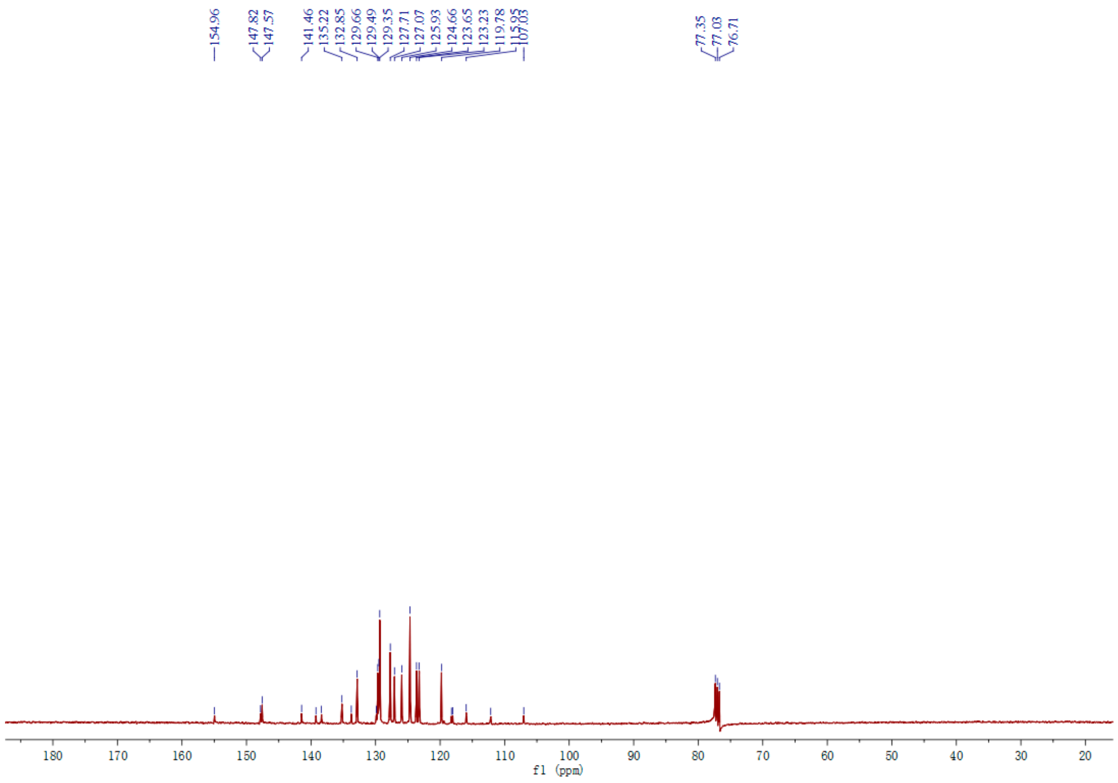

^{13}C NMR spectra of TPA-CDP.

1.7. Figures S1–S6

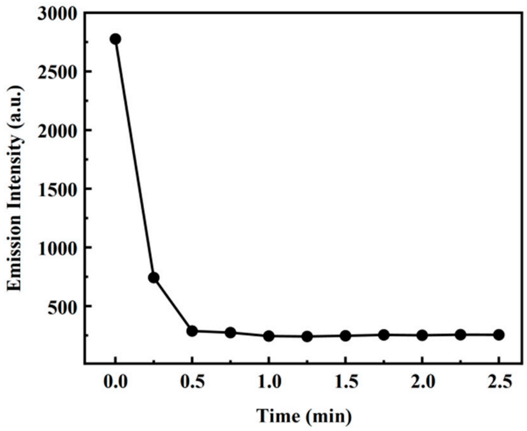

Figure S1. Effect of response time on the fluorescence intensity of TPA-CDP (20μM) in the presence of I^-.

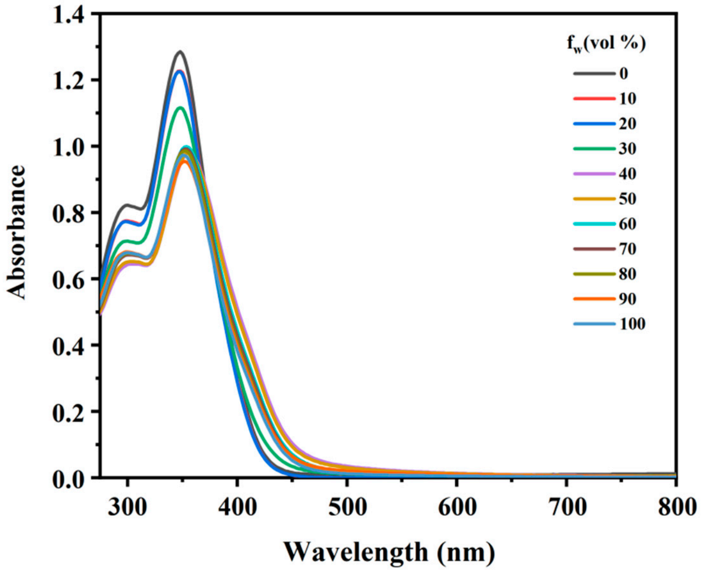

Figure S2. UV–vis spectra of TPA-CDP in DMF–water mixtures of varying water content (0-100%). Concentration: 20 μM.

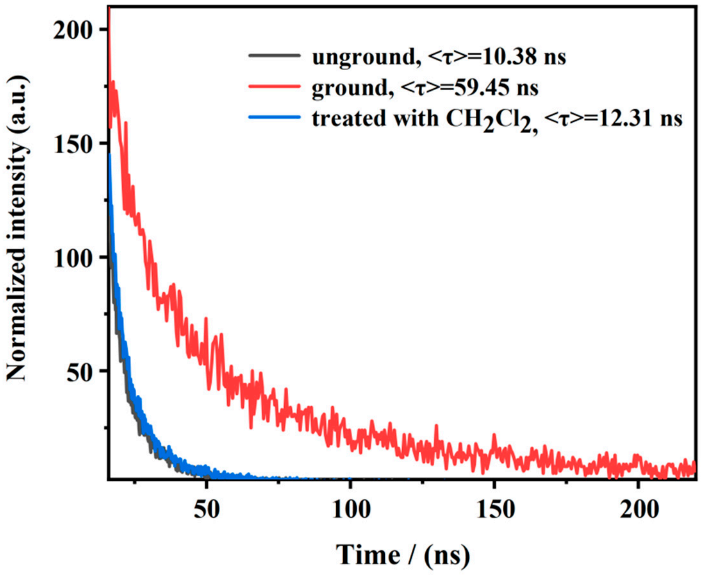

Figure S3. Fluorescence decay curves of unground solid TPA-CDP (345 nm), ground solid TPA-CDP (345 nm), and ground solid TPA-CDP (345 nm) after treatment with dichloromethane.

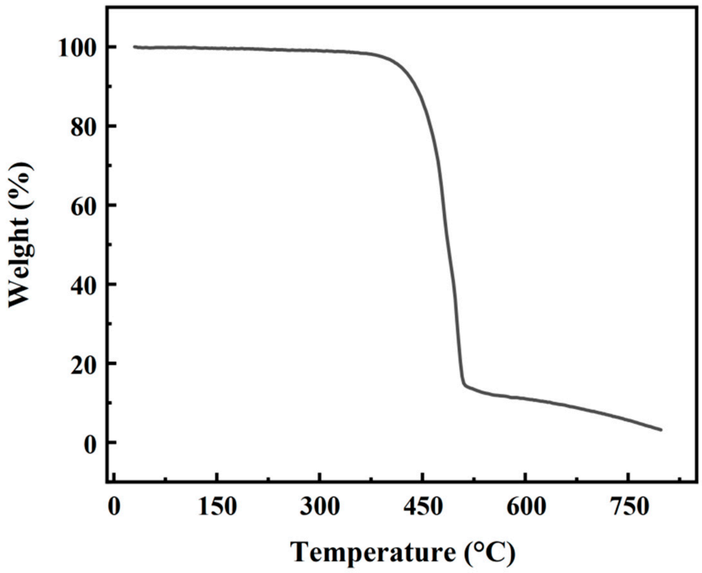

Figure S4. TGA thermograms of solid-state TPA-CDP.

Table 1. The Cartesian coordinates of the optimized geometry.

atom	X	Y	Z	atom	X	Y	Z
C	9.67726	2.95741	−1.19409	C	−5.92403	−2.46611	−0.53717
C	9.97025	2.24328	−0.03041	C	−6.94507	−3.04384	−1.31088
C	9.12948	1.22041	0.40452	C	−8.23931	−3.15821	−0.81597
C	7.98425	0.88343	−0.3343	C	−8.53764	−2.70488	0.47924
C	7.69764	1.59591	−1.50977	C	−7.5181	−2.14762	1.27098
C	8.53453	2.62927	−1.92678	C	−6.22868	−2.03581	0.76609
N	7.12985	−0.16882	0.09975	C	−4.07003	−3.53741	−1.77279
C	5.72372	−0.0076	0.06051	N	−3.71538	−4.48785	−2.34554
C	7.69734	−1.38072	0.58548	C	−9.87064	−2.81509	0.99434
C	8.78014	−1.97591	−0.08083	N	−10.9535	−2.89832	1.4117
C	9.34105	−3.15566	0.40508	H	10.33141	3.75844	−1.5263
C	8.82412	−3.77009	1.54764	H	10.85275	2.49099	0.5537
C	7.74177	−3.18426	2.20755	H	9.35457	0.67721	1.31687
C	7.18609	−1.9949	1.73985	H	6.81914	1.33457	−2.09131
C	4.88567	−1.07854	−0.29484	H	8.29794	3.1701	−2.83941
C	3.50539	−0.91925	−0.32026	H	9.17607	−1.50878	−0.97703
C	2.8993	0.31109	−0.01019	H	10.17882	−3.60336	−0.12312
C	3.74822	1.3778	0.33391	H	9.25935	−4.69342	1.91905
C	5.12898	1.22574	0.37777	H	7.33386	−3.64651	3.10268
C	1.42833	0.4752	−0.04289	H	6.35456	−1.53543	2.26485
C	0.56998	−0.57485	0.32559	H	5.32454	−2.03417	−0.56243
C	−0.81329	−0.4246	0.29176	H	2.88763	−1.75799	−0.62893
C	−1.3969	0.78604	−0.1166	H	3.3184	2.33497	0.61591
C	−0.54355	1.84483	−0.47277	H	5.75566	2.06135	0.67177
C	0.83619	1.68988	−0.43844	H	0.99104	−1.51115	0.67985

C	−2.85549	0.97365	−0.15543	H	−1.44216	−1.24372	0.62771
C	−3.88559	0.02071	−0.48125	H	−0.97951	2.78537	−0.79391
C	−5.06417	0.75141	−0.36331	H	1.46904	2.51385	−0.7552
N	−4.72502	2.01538	−0.01337	H	−6.09072	0.45581	−0.50593
N	−3.38028	2.16416	0.12849	H	−7.21459	2.34091	−0.96786
C	−5.59039	3.11875	0.2314	H	−8.71595	4.23489	−0.47875
C	−6.87642	3.14173	−0.3184	H	−7.93754	6.1186	0.94912
C	−7.71804	4.22023	−0.05013	H	−5.63604	6.0736	1.89576
C	−7.27951	5.27865	0.74691	H	−4.13584	4.14094	1.43379
C	−5.98763	5.25313	1.27668	H	−2.67605	−1.49705	−1.36762
C	−5.139	4.17686	1.02684	H	−6.71871	−3.40836	−2.30837
C	−3.67484	−1.32284	−0.9745	H	−9.0219	−3.59878	−1.42501
C	−4.53796	−2.37499	−1.07299	H	−7.74185	−1.81822	2.28054
H	−5.44425	−1.61983	1.3895				

References

1. He, H.F.; Li, T.; Yao, L.F.; Liu, M.J.; Xia, H.Y.; Shen, L. Aggregation-induced emission enhancement (AIEE)-active donor-acceptor type fluorophores based on the 1,3-diaryl pyrazole unit: Conspicuous mechanochromic fluorescence and biosensing of iodide ions. *Dye. Pigment.* **2022**, *203*, 110309.
2. Ahumada, M.; Lissi, E.; Montagut, A.M.; Valenzuela-Henríquez, F.; Pacioni, N.L.; Alarcon, E.I. Association models for binding of molecules to nanostructures. *Analyst* **2017**, *142*, 2067–2089.
3. Shortreed, M.; Kopelman, R.; Kuhn, M.; Hoyland, B. Fluorescent fiber-optic calcium sensor for physiological measurements. *Anal. Chem.* **1996**, *68*, 1414–1418.
